# Review, Evaluation, and Directions for Gene-Targeted Assembly for Ecological Analyses of Metagenomes

**DOI:** 10.3389/fgene.2019.00957

**Published:** 2019-10-15

**Authors:** Jiarong Guo, John F. Quensen, Yanni Sun, Qiong Wang, C. Titus Brown, James R. Cole, James M. Tiedje

**Affiliations:** ^1^Center for Microbial Ecology, Michigan State University, East Lansing, MI, United States; ^2^Department of Electronical Engineering, City University of Hong Kong, Kowloon, Hong Kong; ^3^Department of Population Health and Reproduction, University of California, Davis, Davis, CA, United States

**Keywords:** gene-targeted assembly, microbial ecology, gene-centric assembly, Xander, MegaGTA

## Abstract

Shotgun metagenomics has greatly advanced our understanding of microbial communities over the last decade. Metagenomic analyses often include assembly and genome binning, computationally daunting tasks especially for big data from complex environments such as soil and sediments. In many studies, however, only a subset of genes and pathways involved in specific functions are of interest; thus, it is not necessary to attempt global assembly. In addition, methods that target genes can be computationally more efficient and produce more accurate assembly by leveraging rich databases, especially for those genes that are of broad interest such as those involved in biogeochemical cycles, biodegradation, and antibiotic resistance or used as phylogenetic markers. Here, we review six gene-targeted assemblers with unique algorithms for extracting and/or assembling targeted genes: Xander, MegaGTA, SAT-Assembler, HMM-GRASPx, GenSeed-HMM, and MEGAN. We tested these tools using two datasets with known genomes, a synthetic community of artificial reads derived from the genomes of 17 bacteria, shotgun sequence data from a mock community with 48 bacteria and 16 archaea genomes, and a large soil shotgun metagenomic dataset. We compared assemblies of a universal single copy gene (*rplB*) and two N cycle genes (*nifH* and *nirK*). We measured their computational efficiency, sensitivity, specificity, and chimera rate and found Xander and MegaGTA, which both use a probabilistic graph structure to model the genes, have the best overall performance with all three datasets, although MEGAN, a reference matching assembler, had better sensitivity with synthetic and mock community members chosen from its reference collection. Also, Xander and MegaGTA are the only tools that include post-assembly scripts tuned for common molecular ecology and diversity analyses. Additionally, we provide a mathematical model for estimating the probability of assembling targeted genes in a metagenome for estimating required sequencing depth.

## Introduction

Metagenomics, involving the shotgun sequencing of DNA extracted from environmental samples, has transformed our understanding of microbial ecology in many environments (Qin et al., 2010; Howe et al., 2014; Sunagawa et al., 2015). This method produces reads from random DNA fragments from genomes in the community (National Research Council, 2007). Thus, it has the potential to both overcome the primer bias issue of amplicon-based methods and to provide a broader functional picture of the sampled microbiome (Frank et al., 2008; Klindworth et al., 2013; Guo et al., 2016). To accomplish this, the reads need to be assembled and/or binned in a meaningful way.

Global assembly and local (targeted) assembly are two common strategies for assembling shotgun reads. Global assembly attempts to recover most if not all genomes in metagenomes and has become a common step for shotgun metagenomic analyses. Many assemblers have been developed for this task including MetaVelvet, IDBA-UD, MEGAHIT, and metaSPAdes (Namiki et al., 2012; Peng et al., 2012; Li et al., 2015; Nurk et al., 2017). While major improvements have been made in recent years, global assembly still faces challenges including repeats, sequencing errors, uneven coverage, and the sheer size of data sets, especially for complex environments such as soil (Li et al., 2015; Nurk et al., 2017; Sczyrba et al., 2017). Many studies, however, only focus on genes involved in certain pathways such as the biogeochemical cycles or other genes that are directly responsible for important ecological functions. In these cases, it is not necessary to assemble all of the shotgun metagenomic data, and local assemblers that target these genes of interest may be more advantageous because they focus computational efforts only on assembly of alleles of a specified gene. In parallel with global assembly, significant progress with local assembly has been made in the last 5 years (Zhang et al., 2014; Wang et al., 2015; Alves et al., 2016; Gregor et al., 2016; Zhong et al., 2016; Huson et al., 2017; Li et al., 2017). This has enabled microbial ecologists to recover full-length (or nearly so) marker genes of phylogenetic or functional interest from complex environmental samples without relying on PCR primers that often amplify only partial gene sequences and have well-known biases (Frank et al., 2008; Klindworth et al., 2013; Guo et al., 2016) resulting in more reliable taxonomic assignments and microbial community diversity analyses. Although the target of local assembly can be any genomic segments including genes, gene cassettes, plasmids, or even whole genomes, we focus on protein-coding gene-targeted assemblers in this review.

There are potential problems with all assembly-based methods. First, the assembled contigs may be chimeric. While some of these can be detected and removed using paired-end information, there is no method to verify all *in silico* (Edgar, 2016). Second, sequence variations from closely related strains are collapsed in the assembly process (Awad et al., 2017; Nurk et al., 2017; Brown et al., 2018). Thus, the assembled contigs are not suitable for SNP (single-nucleotide polymorphism), primer design, or diversity analyses that involve fine taxonomic (species or strain) level discrimination. Third, rare members do not have enough coverage to assemble. All of the above are more problematic in complex metagenomes from environments that have high diversity with many closely related strains and many strains with low coverage (Howe et al., 2014).

Gene-targeted assemblers have potential advantages over global assemblers that may minimize such problems: (1) assembly guided by reference can reduce chimera formation and assembly errors arising from sequencing errors; (2) better efficiency from reduced graph and/or search space enables gene-targeted assemblers to use more sophisticated algorithms to explore micro-heterogeneity of closely related strains (Wang et al., 2015; Huson et al., 2017); and (3) the most common current genome binning approach, which relies on the results from global assembly, misses even more low coverage members than targeted assembly since only bins with high completeness and low contamination are usually selected for downstream analyses (Brown et al., 2018). While many gene-targeted assemblers reviewed here demonstrated better performance than global assembly in their original studies (Zhang et al., 2014; Wang et al., 2015; Huson et al., 2017; Li et al., 2017), continual improvements in global as well as gene-targeted assemblers may result in different performances which may also depend on data size, quality, and gene characteristics. Here, we focus on comparing gene-targeted assemblers rather than gene-targeted assemblers *versus* global assemblers.

While assembly outputs are linear sequences, assembly processes require more sophisticated graph data structures. The two most common data structures are *de Bruijn* graph (DBG) and overlap graph (Myers, 2016). The DBG method first chops reads into even smaller kmers and then builds a graph connecting kmers that share k − 1 bases. The overlap graph method first finds overlaps (larger than a length cutoff) among all reads and then connects reads based on the overlapping information (Peltola et al., 1984; Simpson and Durbin, 2012). Earlier methods for constructing the overlap graph required all-against-all pairwise read comparisons and thus were computationally expensive. Recently, efficient overlap detection methods using advanced data structures such as FM-index and Burrows and Wheeler Transform (Lippert et al., 2005; Simpson and Durbin, 2012) have been developed and make overlap detection highly efficient. The DBG is anti-intuitive by breaking down the reads first, but it achieves faster CPU time by avoiding the expensive all-against all pairwise comparisons since the connections among the kmers are implicit (there are only eight possible neighboring kmers for each kmer by extending A, T, C, or G on both ends). DBG is very sensitive to sequencing errors because each sequencing error can cause k spurious kmers and greatly increase the complexity of the graph. Overall, for global metagenomic assembly the overlap graph works well with long reads by preserving the integrity of the reads, whereas the DBG fits well with the massive amounts of short reads that second-generation sequencing platforms produce (Simpson and Pop, 2015; Myers, 2016).

### Protein-Coding Gene-Targeted Assemblers

Here, we review and compare the efficiencies and assembly quality of several gene-targeted assembly tools: Xander, MegaGTA, SAT-Assembler, HMM-GRASPx, GenSeed-HMM, and MEGAN’s gene-centric assembler (Zhang et al., 2014; Wang et al., 2015; Alves et al., 2016; Huson et al., 2016; Zhong et al., 2016; Huson et al., 2017; Li et al., 2017). Our goal is to give biologists an easy-to-understand review on the gene-targeted assembly algorithms. This is not a complete list of all gene-targeted assemblers. Rather, our selection criteria were (1) unique innovations in assembly algorithms and (2) scalability with large shotgun metagenomic data.

The tools reviewed here use a wide range of algorithms and can be divided into two main categories (**Table S1**): (1) read filtering, potentially iteratively, using sequences or pHMMs as search queries, and (2) assembly by alignment, where pHMMs are used for guiding graph traversal in assembly. Among the tools reviewed, pHMM-GRASPx, GenSeed-HMM, and SAT-Assembler belong to first category. HMM-GRASPx and GenSeed-HMM use iterative read-filtering steps to potentially elongate nascent contigs and then apply third party tools for assembly, while SAT-Assembler has a novel assembly algorithm. MEGAN’s gene-centric assembly function is similar to the first category except that it first aligns all reads against NCBI-nr and subsets reads that align to target genes. Further, Xander and MegaGTA belong to the second category and share a novel pHMM-guided graph traversal algorithm.

#### 1) Xander

Xander combines a DBG with a protein profile Hidden Markov Model (pHMM) built from a reference set of target gene sequences. The probabilities from the pHMM guide gene assembly (Wang et al., 2015). The DBG is encoded as a lossy (approximate) data structure which compresses the sequence data (Pell et al., 2012). The memory needed for this data structure is dependent on the data complexity, not total data size. Xander requires the user to specify the amount of memory before compression. If too little memory is specified for an accurate compression, the user will need to re-run the time-consuming compression. Xander searches start at all nucleotide kmers with sequences that potentially encode short protein sequences found in one or more target gene reference sequences. These starting kmers are extended in both 5’ and 3’ directions using the encoded pHMM probabilities to find high-probability paths in the graph structure, analogous to the way a pHMM is used to find high-probability alignments in a (linear) DNA sequence. The traversal advances three graph nodes (three kmers) at a time (one codon) to select a single reading frame for the pHMM. Xander uses the “A*” algorithm (Hart et al., 1968) to find the path with the highest probability and can also find multiple paths from one start, which is important when studying allelic diversity, using the modified Yen’s K shortest path algorithm (Yen, 1971; Lawler, 1972), which is further modified to require each additional path to contain at least one unique kmer. Therefore, pHMM-guided graph traversal not only reduces the search space compared to global assembly but also provides a probability measure analogous to the familiar BLAST bits score for how likely a contig would have matched the pHMM by chance and thus reduces assembly error.

To assemble sequences, Xander requires forward and reverse pHMMs built from a relatively small set of protein sequences (e.g., 117 for *rplB*) that capture the diversity of the target gene, and a larger set of aligned protein sequences (1,743 for *rplB* but can be several thousands) for finding starting kmers. The current Xander package includes models for the single copy ribosomal protein gene *rplB* and a few N cycle genes (AOA, AOB, *nifH*, *nirK*, *nirS*, *norB_cNor*, *norB_qNor*, *nosZ_cladeI*, and *nosZ_cladeII*). A tutorial is provided for preparing the required pHMMs and references for additional genes (https://github.com/rdpstaff/Xander_assembler#per-gene-preparation-requires-biological-insight). 

Another unique aspect of Xander is that it is designed for microbial diversity analyses and thus includes post-assembly utilities such as chimera checking, *de novo* OTU clustering, taxonomic classification (the nearest neighbor in the reference database with percent identity), and quantification. After assembly, the contigs are clustered at 99% to remove redundancy, and the chimeras are removed by UCHIME (Edgar et al., 2011). For these post-assembly tasks, Xander requires a large set of protein sequences with taxonomy information in the descriptions (usually the same as those used for finding starting kmers) and a comparable set of nucleotide sequences.

#### 2) MegaGTA

MegaGTA is designed based on Xander’s analysis framework and claims several improvements: (1) MegaGTA uses a different space-efficient variant of DBG, the succinct *de Bruijn* graph (sDBG) that was first implemented in the popular global assembly tool MEGAHIT (Li et al., 2015). The sDBG is highly parallelizable and can also be used to build an iterative DBG (Peng et al., 2010), which is difficult to achieve with the bloom filter employed by Xander. The iterative DBG allows the use of multiple kmer sizes, increasing sensitivity and specificity. (2) Xander is designed to remove erroneous kmers caused by sequencing errors by filtering out kmers with low abundance but then keeps single-copy “mercy-kmer” (Li et al., 2015) if they are the only kmers connecting two abundant kmers in a read for the purpose of retaining low abundance species in metagenomes. These are common in complex environments, but this could potentially reintroduce kmers that are sequencing errors. Although pHMM-guided graph traversal should reduce the chance of erroneous kmers entering assemblies, MegaGTA does penalize kmers with low coverage in the guided assembly step. This reduces assembly error from sequencing errors but might also introduce bias against low abundant members. Overall, MegaGTA achieves better sensitivity and specificity, although its memory requirement can still be a hindrance for large and complex metagenomes.

#### 3) SAT-Assembler

Similar to Xander and MegaGTA, SAT-Assembler also uses pHMM, but it is a string graph–based assembler that includes two main steps. The first step searches for target gene fragments in reads using pHMM with HMMER3 with a permissive cutoff (e-value cutoff of 1,000), which greatly reduces the input data size for the next step without losing sensitivity. The second step builds a string graph for each targeted gene and assembles contigs. The read alignment location information against the model from the first step is used to guide the overlap calculation among reads. Multiple types of information such as paired ends, overlap connection, and coverage are used to guide graph traversal and avoid chimeras. Contigs are merged into scaffolds using paired-end information as the final step. To run SAT-Assembler, a file containing pHMMs of targeted genes is required. The pHMM for a specific gene can be built from aligned protein sequences of the gene using the hmmbuild command in HMMER3 (Eddy, 2009). Additionally, SAT-Assembler is also designed to work with pHMMs in the Pfam database, which has ∼ 18,000 pHMMs in version 32.0 and covers ∼ 80% of protein sequences in UniProtKB (Finn et al., 2016; Schaeffer et al., 2017).

As mentioned briefly above, Xander/MegaGTA and SAT-Assembler use pHMMs in very different ways. In Xander/MegaGTA, pHMMs are used to guide graph’s traversal in DBG. Although the graph traversal space is reduced to those paths related to the target gene, it is still computationally expensive (CPU time and memory) to load all reads into the graph and identify all starting kmers in a large graph. In contrast, SAT-Assembler uses pHMMs to filter reads belonging to target genes as a data reduction step and then uses the reduced dataset to build the assembly graph, thus greatly reducing the memory and CPU cost of graph building. SAT-Assembler further uses read pHMM alignment information to speed up overlap computation among reads for building string graphs. It, however, does not apply pHMM to guide graph traversal on the resulting string graph, which could potentially improve the assembly.

#### 4) HMM-GRASPx

HMM-GRASPx is also pHMM-based, but it integrates many tools including gene callers (MetaGeneAnnotator/FragGeneScan) (Noguchi et al., 2008; Rho et al., 2010), HMMER3 (Eddy, 2009), nucleotide sequence assembler (SPAdes) (Nurk et al., 2017), and protein sequence assembler (SFA-SPA) (Yang et al., 2015). Its core algorithm, iterative search and assembly, is based on an overlap graph in protein space and hence can increase the sensitivity of gene identification. Short reads are not ideal for gene identification because they may not have enough information to be recognized as the target gene. HMM-GRASPx tackles this problem by iterative search and assembly. Intuitively, homologous protein sequences translated from reads with low sequence identity could be identified by being assembled first with other high identity reads into longer contigs. More specifically, (1) overlaps among reads are firstly computed, (2) reads with high pHMM alignment scores are identified and used as starting contigs, (3) contigs are extended using overlapping reads, and (4) the extended contigs are aligned with pHMM to decide whether to continue extending. If the alignment score is below a certain threshold or there are no more overlapping reads, then the extension stops; (5) the resulting contigs are assembled again based on their overlap; and (6) finally, reads from the target gene are retrieved by mapping them to the assembled gene contigs. This core algorithm functions both as a finder and assembler. HMM-GRASPx’s authors suggest that, for quantitative results, the identified contigs be assembled with another program, i.e., SPAdes for nucleotide and SFA-SPA for protein reads. This is because the algorithm outputs all possible contigs to increase sensitivity and thus can produce redundant assemblies. However, it should be possible to simply remove the redundant contigs, which would improve the overall computational efficiency.

#### 5) GenSeed-HMM

GenSeed-HMM applies an iterative assembly and extension strategy similar to that used by HMM-GRASPx. The key difference is GenSeed-HMM can extend beyond the gene boundaries, while HMM-GRASPx will automatically stop extending when the pHMM alignment score drops. GenSeed-HMM has the advantage of being able to use nucleotide, protein sequences, or pHMMs as references, which gives the users more flexibility. Internally, it applies BLASTn with nucleotide references and TBLASTN for protein references to search against the (nucleotide) reads, and hmmsearch for pHMM search of the translated reads. At the assembly step, it uses third party assembly tools such as SOAPdenovo, ABySS, and CAP3 (Huang and Madan, 1999; Simpson et al., 2009; Li et al., 2010; Luo et al., 2012), and the choice of third party assembly tools might have an impact on its overall computational efficiency and assembly quality. For contig extension iterations, contig ends are extracted and used as new references for the next search iteration. If no contigs are extended, it will trim the extended part from the previous iteration and try new extension up to three iterations. Once a contig reaches or exceeds the maximum length set, it will not be included in subsequent iterations. GenSeed-HMM is not a typical gene-targeted assembler since its contigs may extend beyond gene boundaries. This makes it useful to study the nearby genes (genomic context) of the target gene. For marker gene–based microbial diversity studies, however, the parts beyond the gene boundaries would have to be trimmed before further analyses.

#### 6) MEGAN-Assembler

MEGAN assembler is part of MEGAN version 6 (Huson et al., 2016; Huson et al., 2017), and its key algorithm is protein alignment-guided assembly, an overlap graph–based method. It requires an all against all pairwise alignment of query metagenomes and reference database such as NCBI-nr using BLAST or DIAMOND (Altschul et al., 1997; Buchfink et al., 2015) as the first step, the same as all other analyses in MEGAN. MEGAN utilizes the above alignment information to find the overlap among reads based on their alignment to the same target references and further constructs overlap graphs based on 100% sequence match in the overlapped portion of the alignment. In this way, MEGAN avoids the expensive computation of all against all comparisons among query reads for constructing overlap graphs (similar to SAT-Assembler). Further, MEGAN weights overlap graph edges (connection between reads) by overlap sizes and then traverses the graph by finding an acyclic path with a maximum weight. It reports contigs with a minimal length, removes the reads used for the assembled contigs in overlap graphs, and iterates the above process until no more paths remain. Contigs are further extended if two contigs have overlap and an overlap identity larger than a certain thresholds (by default 20 bp and 98%, respectively). Although inducing the read overlap from alignment against references is a good strategy to improve computational efficiency, the first step of all *vs*. all comparison of query to NCBI-nr is still a daunting task for large metagenomes.

## Methods

### Data

We evaluated the performance of these gene-targeted assemblers using three data sets. The synthetic data consisted of 150-bp single reads without errors generated from the 17 genomes in **Table S2** using Grinder (Angly et al., 2012) with the parameters “-rd 150 -cf 10” to give 10X coverage of each genome. The seven species of *Pseudomonas* were selected as a challenge for assemblers regarding their production of chimeric contigs. The mock community data, generated from a mixture of known amounts of gDNA from 16 archaeal and 48 bacterial strains (Shakya et al., 2013), consisted of 100-bp paired Illumina reads downloaded from NCBI as run SRR606249. These reads were trimmed using fastq-mcf (version 1.04.662) (http://code.google.com/p/ea-utils) with the parameters “-q 30 -l 50 -w 4 -x 10 -max-ns 0 -X.” The soil metagenome sample was sample C1 that was included in the original Xander paper (Wang et al., 2015) and is available from NCBI as run SRR3989263. Fifty million reads sampled from C1 were trimmed with fastq-mcf with the same parameters above and converted to FASTA format to give 33.7 million paired reads designated C1-50M.

### Programs

Xander is included in RDPTools, which is available as source on GitHub (https://github.com/rdpstaff/RDPTools). It requires Python 2.7+, Java 1.6+, HMMER 3.1 (http://hmmer.janelia.org), and UCHIME (http://drive5.com/usearch/manual/uchime_algo.html). All of these dependencies may be met by instead installing the Bioconda package from https://bioconda.github.io/recipes/rdptools/README.html. Instructions for Xander are available at https://github.com/rdpstaff/Xander_assembler and https://john-quensen.com/workshops/workshop-2/xander. We installed RDPTools from source. All required reference files for *rplB*, *nifH*, and *nirK* are included in the installation.

Two of Xander’s parameters depend on the input file size. We set FILTER_SIZE to 32, 36, and 38, and MAX_JVM_HEAP to 4G, 12G, and 64G for the synthetic, mock, and C1-50M data, respectively. We set MIN-COUNT to 1 and left all other parameters at their default values for all cases. Resulting false-positive error rates were always less than 3.20E−05.

MegaGTA is a re-write in C++ of the first two portions of Xander: build and find. It may be installed from source from https://github.com/HKU-BAL/megagta or as a Bioconda package from https://bioconda.github.io/recipes/megagta/README.html. MegaGTA requires RDPTools. If installed from source, RDPTools is included. If installed from Bioconda, RDPTools must be installed separately. We installed the Bioconda package.

We limited the available memory for MegaGTA to 19.2G for the synthetic data and left all other parameters at their default values, including memory, for the other data sets. Memory is set as a fraction (0.8 by default) of available memory. The gene_list.txt configuration file used pointed to the for_enone.hmm, rev_enone.hmm, and ref_aligned.fasta files for each gene (*rplB*, *nifH*, and *nirK*) in the RDPTools/Xander_assembler/gene_resource directory.

We installed SAT-Assembler from the forked version on GitHub at https://github.com/jiarong/SAT-Assembler, following the instructions on that web page. Older versions of SAT-Assembler on SorceForge.net and at https://github.com/zhangy 72/SAT-Assembler no longer work because of updates to some of the modules the program requires. For this program, HMM-GRASPx and GenSeed-HMM, we used pHMMs downloaded from the FunGene web page (http://fungene.cme.msu.edu/).

We installed HMM-GRASPx from https://sourceforge.net/projects/hmm-graspx/ and followed the directions under the Files tab on that page. To generate input files for HMM-GRASPx, we ran FragGeneScan with parameters “-complete 0 -train illumine_5 –thread 4.” For HMM-GRASPx, we left all parameters at their default values.

We installed the Linux version of MEGAN and its auxiliary mapping files from http://ab.inf.uni-tuebingen.de/data/software/megan6/download/welcome.html. Use of MEGAN for gene-centric assembly from metagenomic data requires that all sequences are first aligned against NCBI’s non-redundant protein database (NCBI-nr). We used DIAMOND (Buchfink et al., 2015) (https://github.com/bbuchfink/diamond) because of its speed and output format 100 since the resulting daa (DIAMOND alignment archive) files are more rapidly imported into MEGAN. We “meganized” the data files using the protein accession to InterPro mapping file acc2interpro-June2018X.bin downloaded from the MEGAN site and the command line tool daa-meganizer. For both DIAMOND and MEGAN assembler, we used the default values for all parameters.

GenSeed-HMM is a Perl script available at https://sourceforge.net/projects/genseedhmm/. It operates by making calls to a variety of third-party tools including BLAST+, hmmsearch, EMBOSS, bowtie, and at least one assembler. We used the ABySS assembler for all of our tests with this program. We used Conda to create an environment containing these programs and their dependencies and ran GenSeed-HMM from within this environment. An YML file for creating the same environment is available at https://github.com/jfq3/Virtual-Environments.

### Assembly Quality

We evaluated two aspects of assembly quality: (1) contigs should capture all target gene sequences known to be in the data (sensitivity), and (2) contigs should not include irrelevant sequences (specificity). Both aspects were evaluated by conducting a BLAST search of contigs against target gene sequences extracted from the genomes, or in the case of the soil sample C1-50M against NCBI-nr. Sequence similarity was defined as “alignment length” * identity/“length of shorter sequence.” Some contigs were too different from the target sequences to appear in the BLAST results. The relationships of such contigs to the target genes were investigated by searching against NCBI-nr and/or against the genomes themselves and viewing the alignment in NCBI’s genome browser. Potentially chimeric sequences assembled from the synthetic and mock data were also flagged by UCHIME using target gene sequences extracted from the genomes as the reference.

To make these tests comparable among assemblers, we compared comparable contigs. For Xander and MegaGTA, we used the intermediate file “_prot_merged_rmdup.fasta.” Post-assembly *per se*, Xander and MegaGTA outputs are normally processed through a pipeline that removes potential chimeras and short sequences and clusters the remaining sequences at a user-defined distance, thus decreasing sequence variation in their final outputs. The file “_prot_merged_rmdup.fasta” has not been subjected to these processes and contains all unique contigs assembled. To investigate chimeras produced by Xander and MegaGTA, corresponding nucleotide sequences were selected from the “nucl_merged.fasta” files; these files are all nucleotide contigs assembled. As well as testing SAT-Assembler and GenSeed-HMM output directly, we also removed duplicate sequences and filtered to a minimum length of 450 bp (using RDPTool’s rm-dupseq command) to produce results more comparable to Xander’s and MegaGTA’s “_prot_merged_rmdup.fasta” files. We also compared MEGAN results filtered to the same minimum length.

### Sequencing Depth Estimation

In genome sequencing, the relation between sequencing depth and genome coverage is already a well-studied problem. Lander–Waterman statistics (Lander and Waterman, 1988) show that with “*L*” as read length, “*N*” as number of reads, and “*G*” as genome length (much larger than read length), the average coverage of genome (“*a*”) is “*LN/G*,” and the probability of each base not being covered (“*p*”) is “*e* *^−a^*.” In the context of metagenomics, however, a targeted species is only “*R*” (relative abundance) of the total community, so “*a*” (the average coverage of genome) should be redefined as “*LNR/G*” (we assume that all species have the same genome size, “*G*,” to simplify the problem). We can further deduce that the probability (“*P*”) of at least “*M*” continuous positions (a contig with at least “*M*” bp) in a target gene with a size of “*S*” bp being covered is:

$$P=\sum_{i=M}^{S}(S-i+1)p^{S-i}{\left(1-p\right)}^{i}$$

Further, the above only considers whether a position is covered but not the read overlaps that are needed for assembly. In DBG graph with kmer size of “*k*,” the minimal overlap required for two reads to connect is “*k − 1*.” To account for the “*k − 1*” overlap in either DBG or overlap graph, we can simply define the effective read to be the first “*L−(k − 1)*” position of each read, so when one shortened read follows right after where a preceding one ended, they effectively have an overlap of “*k − 1*.” Therefore, “*p*” can be redefined as the probability of a position not being covered by reads of effective length (“*L − k + 1*”) with the value:

$$p=e^{-(L-k+1)NR/G}$$

To evaluate the effect of sequencing depth on gene-targeted assembly, we first evenly divided our soil metagenome (C1) into 2, 4, 8, 16, and 32 subsamples. For each sample, we ran Xander to assemble *rplB* with the same parameters mentioned above. The coverage information was retrieved from mean kmer coverage in “_rplB_45_coverage.txt” output file. We also included *rpsC* as a confirmation of *rplB* results. The reference files of *rpsC* for Xander can be downloaded from http://doi.org/10.5281/zenodo.1410823 (Guo, 2018).

## Results

### Time and Memory Requirements

Comparisons of computer time and memory resources required are complicated by the programs having different prerequisites and end points. Overall, SAT-Assembler was the most efficient requiring less than 6-min wall time and only 78 MB of memory to process the synthetic data for *rplB* (**Table 1**). SAT-Assembler stops short of providing quantitative results allowing sample comparisons as Xander does; such further processing would be close to that for MegaGTA’s post-processing step. Xander’s three steps took only slightly longer (7 min 31s) to provide quantitative results but required approximately 1.5 GB of memory. Xander’s build step is considered a bottleneck because it is not multithreaded, and MegaGTA is advertised as advancement over Xander in part because of greater speed. This is true only for wall time and if enough threads are used; the actual CPU time (78 min) was much greater than Xander’s but did require slightly less memory. The memory requirement for GenSeed-HMM was comparable to that of Xander, but the processing time was approximately twice as long without including any of the post-processing steps required for making sample comparisons.

**Table 1:** Time and memory requirements for processing the synthetic data for *rplB*. Except for MEGAN BLAST/DIAMOND performed on MSU’s cluster, all times are for running on an HP ProBook 450 G5 with Intel i7-8550U CPU and 32 Gb RAM running Ubuntu 18.04 LTS.

Program	Stage	Threads	Wall timehh:mm:ss	CPU timehh:mm:ss	Peak memory (KB)
Xander	Build	1	00:03:52	00:03:57	736,860
	Find	4	00:00:57	00:04:48	1,512,728
	Search	4	00:02:42	00:04:28	867,776
MegaGTA	Main	8	00:10:06	01:15:02	1,133,248
	Post-processing	4	00:00:47	00:02:16	729,624
FragGeneScan		4	00:24:20	01:29:15	65,356
HMM-GRASPx		4	00:05:28	00:05:28	8,159,504
SAT-Assembler		NA	00:05:55	00:06:38	77,620
MEGAN	Diamond	8	14:38:57	95:11:48	19,810,188
	Meganize	NA	00:05:46	00:15:57	21,659,968
	Assembly	NA	00:00:03	NA	NA
GenSeed-HMM		4	00:07:46	00:16:57	1,425,368

The pre-processing required by HMM-GRASPx and MEGAN made them much less efficient to implement. HMM-GRASPx requires that all fragments first be translated into peptide reads by FragGeneScan or MetaGeneAnnotator. Furthermore, to obtain accurate quantitative results, the authors recommend that the contigs be re-assembled by another program; time and memory requirements for that process are not included in **Table 1**. MEGAN is by far the least efficient, requiring that all fragments first be aligned against NCBI’s non-redundant protein database. For this task, DIAMOND is preferred over BLAST due to its much greater speed (still required over 95-h CPU time), but the speed comes with a higher memory requirement (20 GB).

### Assembly Quality Tested With Synthetic Data

GenSeed-HMM was the most successful at capturing exact matches to the *rplB* genes in the synthetic data, matching all 17 with 100% identity (**Table 2**). HMM-GRASPx, MEGAN, and SAT-Assembler did nearly as well, matching 16 of the sequences at 100% identity. HMM-GRASPx missed *Pseudomonas putida* while MEGAN missed *Lacunisphaera limnophila *even at a lower 97% identity threshold. Many of the exact matches produced by HMM-GRASPx, MEGAN, and GenSeed-HMM were short; however, they captured only about half of the target genes if comparisons were restricted to contigs of at least 450 nucleotides. Xander and MegaGTA were the worst at producing exact matches, capturing only 12 of the 17 genes at 100% identity.

**Table 2:** BLAST summary for *rplB* assembled from the synthetic data. There were 17 *rplB* sequences in the synthetic data. Entries in the % ID columns give the number of taxa matched over the number of contigs that match *rplB* by BLAST identity at the specified percentage.

Method	Contigs	Length	Non-target	<97%	97%	98%	99%	100%
Xander	28	807–828	0	1	17/27	15/23	12/16	12/12
MegaGTA	28	807–828	0	1	17/27	15/23	12/16	12/12
HMM-GRASPx	63	102–261	0	3	16/60	16/60	16/59	16/59
HMM-GRASPx	0	> =450	–	–	–	–	–	–
MEGAN^1^	55	204–3,822	32	0	16/23	16/23	16/23	16/23
MEGAN^2^	20	453–3,822	11	0	9/9	9/9	9/9	9/9
SAT-Assembler^3^	176	150–997	49	60	17/67	17/50	16/28	16/23
SAT-Assembler^4^	106	465–997	0	58	16/48	15/33	13/14	11/11
GenSeed-HMM^5^	97	32–1,340	4	0	17/93	17/93	17/93	17/93
GenSeed-HMM^6^	9	724–1,340	1	0	8/8	8/8	8/8	8/8

MEGAN^1^: all contigs assembled. MEGAN^2^: contigs filtered to a minimum length of 450 bp. SAT-Assembler^3^: all contigs assembled with an overlap length of 40 bp. SAT-Assembler^4^: contigs were de-replicated, duplicates removed, and filtered to a minimum length of 450 bp. GenSeed-HMM^5^: all contigs assembled; GenSeed-HMM^6^: contigs were filtered to a minimum length of 450 bp.

These same two assemblers were the best, however, at excluding irrelevant sequences; all 28 contigs were at least 96% identical to *rplB* gene sequences, and all 17 taxa were captured at a 97% identity threshold. HMM-GRASPx also did well, with only 5% of its assemblies having BLAST matches to *rplB* of less than 97% identity. MEGAN, on the other hand, assembled 32 contigs (58% of the total) that were perfect matches to portions of the reference genomes but entirely unrelated to *rplB*, and 58 to 60% of the SAT-Assembler assemblies had less than 97% identity to *rplB* genes in the synthetic data. GenSeed-HMM also assembled some sequences unrelated to the target sequences.

Except for SAT-Assembler, all tools assembled contigs matching all six *nifH* (nitrogenase reductase) sequences present in the synthetic data with at least 97% identity (**Table S3**). SAT-Assembler did not match any of the reads to *nifH* and so did not assemble any contigs for the gene. MEGAN and GenSeed-HMM also produced high proportions of contigs (11 of 20 and 71 of 127, respectively) unrelated to nifH sequences in the synthetic data.

HMM-GRASPx, MEGAN, SAT-Assembler, and GenSeed-HMM all assembled contigs with 100% identity to all four *nirK* (nitrite reductase) sequences present in the synthetic data (**Table S4**). Xander and MegaGTA performed identically, each producing contigs which matched only two of the *nirK* sequences present in the synthetic data, but with 100% identity. MEGAN, SAT-Assembler, and GenSeed-HMM again produced non-relevant contigs.

### Assembly Quality Tested With Mock Data

Overall, MegaGTA was the most successful at assembling *rplB* contigs from the mock data, producing 86 unique contigs of more than 450 bp with at least 97% identity to 46 of the 48 bacterial *rplB* sequences present (**Table 3**). While SAT-Assembler using an overlap length of 40 produced more (1,318) contigs with 100% identities to 47 of the 48 rplB sequences present, most of the contigs were very short. There were only 61 unique contigs of at least 450 bp, and only 13 of these matched expected *rplB* sequences with 100% identity. Xander did nearly as well as MegaGTA, while for MEGAN’s contigs, over 450 bp matched only 33 of the *rplB* sequences with at least 97% identity and GenSeed-HMM’s matched 28 with 100% identity. All the assemblers produced “missing” contigs, i.e., ones that did not appear in the BLAST tables due to very low sequence similarity to reference sequences. By BLAST to NCBI-nr, all of these produced by Xander, MegaGTA, and SAT-Assembler matched known *rplB* sequences at more than 99% identity. Only one, however, of the 45 produced by MEGAN was related to *rplB*.

**Table 3:** BLAST summary for *rplB* contigs assembled from the mock data. There were 48 bacterial *rplB* sequences in the mock data set. Entries in the % ID columns give the number of taxa matched over the number of contigs that match *rplB* by BLAST identity at the specified percentage.

Method	Contigs	Length	Non-target	<97%	97%	98%	99%	100%
Xander	95	459–849	2	5	44/88	43/85	40/80	30/30
MegaGTA	94	453–849	2	6	46/86	44/83	42/80	32/32
MEGAN^1^	93	201–1,611	45	1	39/47	39/47	38/46	35/39
MEGAN^2^	50	450–1,611	16	1	33/33	33/33	32/32	28/28
SAT-Assembler^3^	2,765	50–750	751	107	48/1,907	48/1,865	48/1,689	47/1,318
SAT-Assembler^4^	61	458–750	1	18	29/42	27/37	25/31	13/13
GenSeed-HMM^5^	408	31–1,360	60	7/9	47/339	47/330	46/187	43/183
GenSeed-HMM^6^	44	450–1,360	11	1/1	28/32	28/32	27/31	23/27

^1^Data for all MEGAN contigs assembled from reads mapping to IPR005880 using default parameters. ^2^Data for MEGAN contigs filtered to a minimum length of 450 bp. ^3^All SAT-Assembler rplB contigs assembled from the mock data with an overlap length of 40 bp. Notice that the minimum length is one-half of the read length. ^4^SAT-Assembler contigs were assembled with an overlap length of 40 bp, de-replicated, duplicates removed, and filtered to a minimum length of 450 bp. HMM-GRASPx failed to complete with this data set. GenSeed-HMM^5^: all contigs assembled; GenSeed-HMM^6^: contigs were filtered to a minimum length of 450 bp.

GenSeed-HMM and MEGAN did slightly better than Xander and MegaGTA in capturing *nifH* sequences in the mock data (**Table S5**), but both again produced high proportions of unrelated contigs and many of GenSeed-HMM’s were very short. As with the synthetic data, SAT-Assembler did not match any of the reads to *nifH* and so did not assemble any contigs for the gene.

SAT-Assembler did assemble *nirK* contigs, matching all five sequences present in the data at 100% identity (**Table S6**), but again, most contigs were short. Only two were over 450 bp, and these matched only one of the five *nirK* sequences in the mock data. GenSeed-HMM did better, producing contigs matching all five target genes with 100% identity even after they were filtered for length, but also a high proportion of contigs unrelated to the *nirK* sequences in the data. MEGAN contigs matched four of the five at 100% identity but also produced a high proportion of unrelated sequences. MegaGTA and Xander produced three and two contigs, respectively, matching two of the target sequences.

### Assembly Quality Tested With Soil Metagenome

For the C1-50M shotgun data, GenSeed-HMM produced the most contigs and matched the highest number of *rplB* sequences in NCBI-nr (**Table 4**). But most of the contigs were very short such that over 70% did not match *rplB* with an e-value of less than 10. Only two were over 450 bp. Considering only contigs over 450 bp, MegaGTA produced the most (316), all of which matched *rplB* sequences in NCBI-nr, and Xander was a close second. MEGAN produced far fewer contigs (30), only 3 of which were over 450 bp, and 11 of which were not *rplB*.

**Table 4:** BLAST summary for bacterial *rplB* contigs assembled from C1-50M aligned against NCBI-nr. Entries in the % ID columns give the number of taxa matched over the number of contigs that match *rplB* by BLAST identity at the specified percentage.

Method	Contigs	Length	Non-target	<97%	97%	98%	99%	100%
Xander	269	453–825	0	56/250	11/19	8/16	4/8	3/3
MegaGTA	316	450–825	0	82/290	13/26	12/19	8/11	4/4
MEGAN^1^	30	207–705	11	2/2	14/17	11/14	9/12	9/12
MEGAN^2^	3	462–705	1	2/2	2/2	2/2	2/2	2/2
SAT-Assembler^3^	705	51–436	9	125/207	179/469	154/381	132/316	131/312
SAT-Assembler^4^	0	–	–	–	–	–	–	–
GenSeed-HMM^5^	4340	31–1,058	3109	334/596	311/635	284/562	277/535	273/535
GenSeed-HMM^6^	4	458–1,058	0	2/2	2/2	2/2	1/1	1/1

MEGAN^1^: all contigs assembled. MEGAN^2^: contigs filtered to a minimum length of 450 bp. SAT-Assembler^3^: contigs assembled with an overlap length of 40 bp and de-replicated. SAT-Assembler^4^: contigs assembled with an overlap length of 40 bp were de-replicated and filtered to a minimum length of 450 bp. GenSeed-HMM^5^: all contigs assembled; GenSeed-HMM^6^: contigs were filtered to a minimum length of 450 bp.

### Chimera

The synthetic data set was meant to be challenging with regard to chimera formation, especially for *rplB*. Xander, MegaGTA, and SAT-Assembler all produced high proportions of *rplB* chimeras from this data set (**Table S7**). For the first two, chimeras were almost exclusively (10 of 11, over 90%) between species of *Pseudomonas*. For SAT-Assembler, however, approximately one fourth of the chimeras were between different genera, and the proportion of chimeras increased with contig length. None of MEGAN’s or GenSeed-HMM’s contigs were flagged as chimeras.

The same trend held for the mock data (**Table S8**). Xander and MegaGTA produced fewer *rplB* chimeras than SAT-Assembler, and when they occurred, they were exclusively between species of the same genus. In contrast, approximately 30 to 40% of the chimeras (depending on length) produced by SAT-Assembler were between different genera. As with the synthetic data, none of MEGAN’s *rplB* contigs were flagged as chimeras, and only 1 of 408 produced by GenSeed-HMM was a chimera.

Xander and MegaGTA also produced a high percentage of *nifH* chimeras from the synthetic data (**Table S9**), but exclusively between sequences from the same genus. In fact, for Xander, two of the five and for MegaGTA three of the five chimeras were between *nifH* copies within the same species. There were only two other instances of chimera formation. Xander formed a *nifH* chimera with the mock data between strains S2 and C5 of *Methanococcus maripaludis*, and MEGAN formed a *nifH* chimera between *Azotobacter vinelandii* and *A. chroococcum*. There were no *nirK* chimeras from either data set by any of the assemblers.

### Sequencing Depth

**Figure 1:**
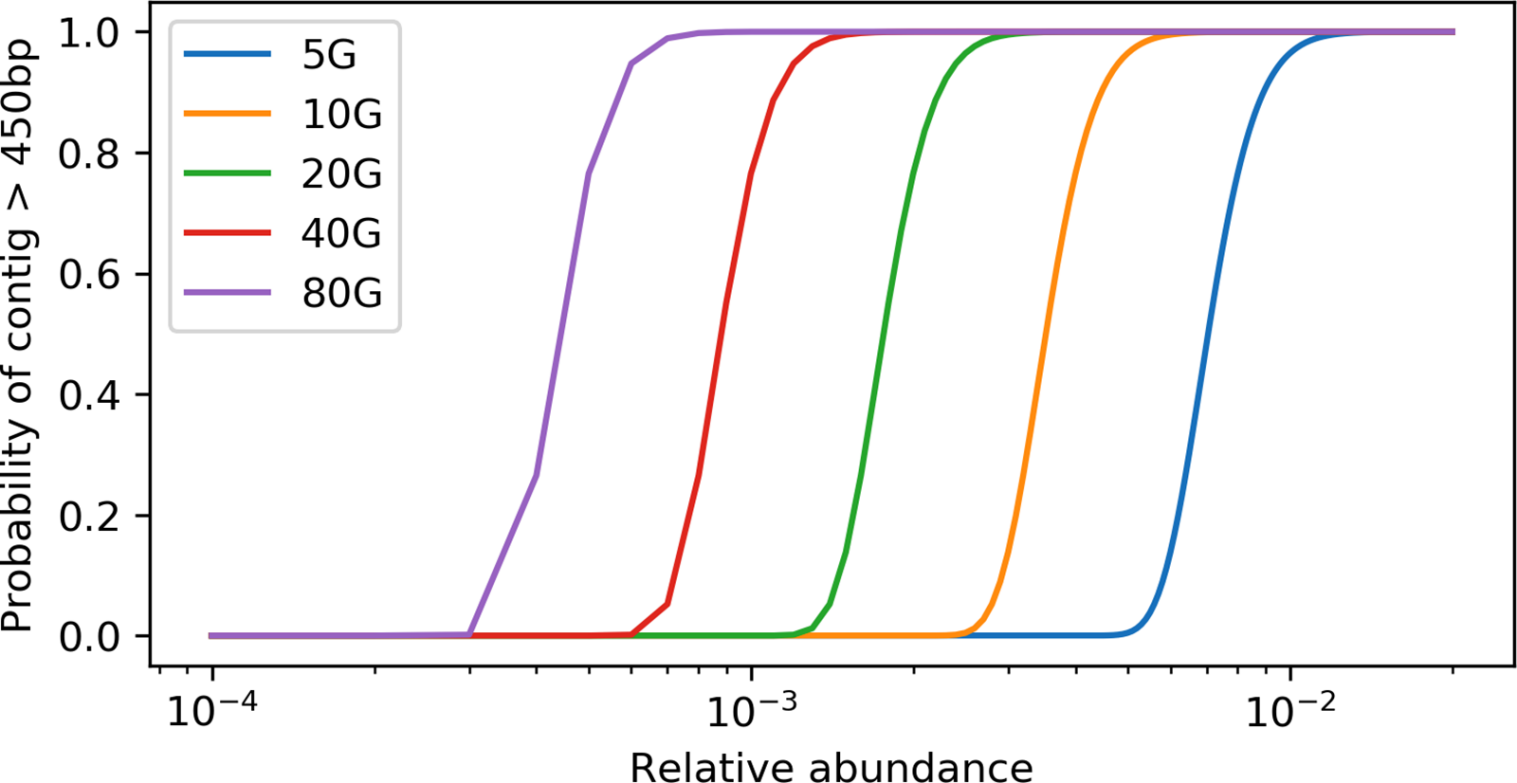
Relation between the probability of having a target gene from a species assembled and the relative abundance of the species at different sequencing depth. X axis is at log10 scale, the target gene length is set to 800 bp, and the minimum contig length is set to 550 bp.

**Figure 2:**
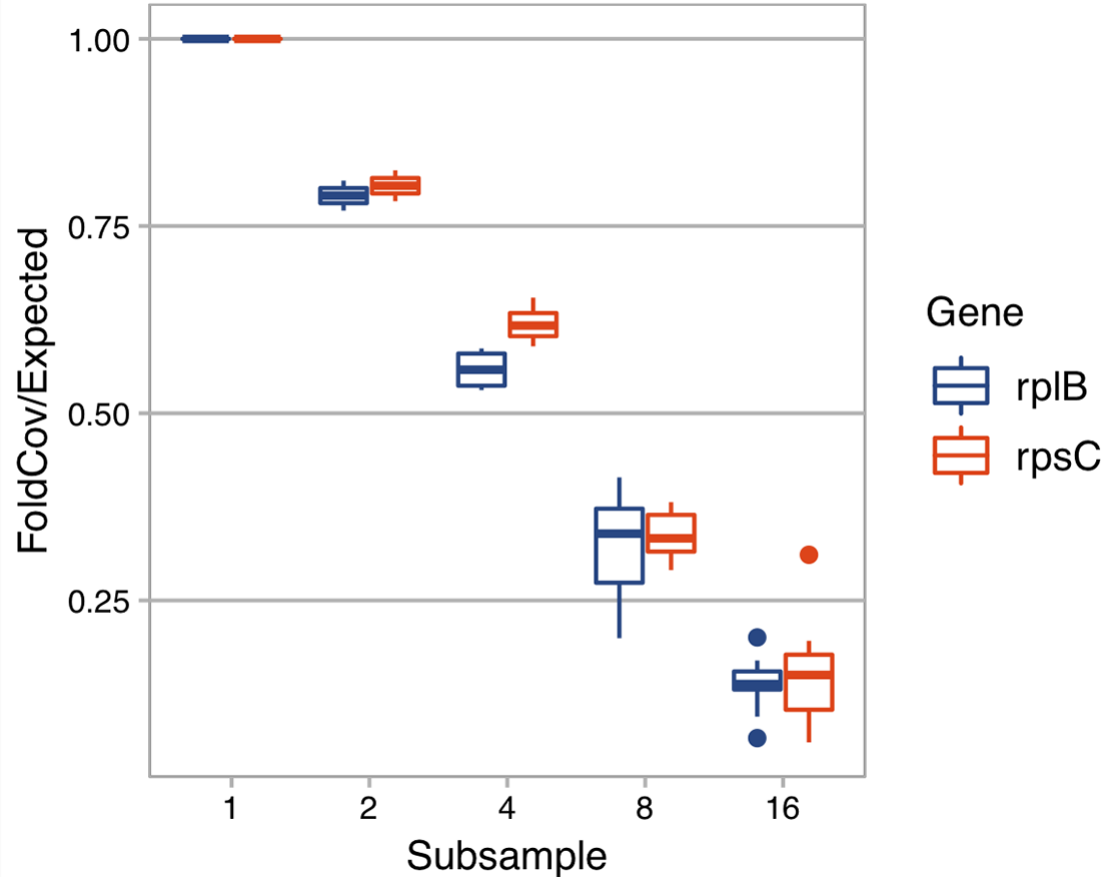
The effect of sequencing depth on the fold coverage of *rplB* or *rpsC* assembled. X axis is the number of subsamples C1 is evenly divided into. Y axis is *rplB* or *rpsC* fold coverage of a subsample divided by expected folded coverage as if it decreases linearly with sequencing depth (the fold coverage of original sample divided by number of even subsamples).

With the derived model, we estimated that ∼ 40 Gbp of sequences is needed to assemble a contig (>450 bp) from a gene with a length of 800 bp in a species that is 0.1% of the metagenome (assuming all genome sizes are 5 Mbp) (**Figure 1**). Additionally, when we evaluated the effect of sequencing depth on assembly by subsampling, we found the number of genes assembled decreased much faster than sequencing depth for both *rplB* and *rpsC* (**Figure 2**).

## Discussion

Computer time and memory requirements can be limiting factors in deciding a method to process metagenomic data. SAT-Assembler required the least time and memory because it first selects a limited number of reads related to the target gene to assemble. HMM-GRASPx employs a similar strategy to reduce time and memory requirements, but by relying on FragGeneScan as a pre-step, it requires far more total time. Furthermore, its pHMM alignment at each contig extension is also computationally expensive and slows down the simultaneous search and assembly step. Similarly, GenSeed-HMM bogs down trying to extend both ends of the numerous sequences it finds in a first pass through complex data, and MEGAN’s reliance on conducting a BLAST search of all sequences against NCBI-nr makes it computationally very expensive to implement. We were only able to compare assembler performance with an environmental sample by reducing the C1 sample to 50 million reads. The full sample is five times as large, and neither GenSeed-HMM nor DIAMOND BLASTX finished processing the full C1 sample within the 7-day limit on our cluster. By contrast, Xander processing of the full C1 data set, including all post-assembly processing, for all three genes considered here took only 18 h 13 min of wall time (40 h 30 min of CPU time).

SAT-Assembler’s savings in resource cost comes at great expense in performance, notably in the production of mostly short contigs. The similarity search step may have missed remote homologs of the references in pHMM despite the loose cutoff used in hmmsearch. Thus, by selecting relatively few reads to assemble, there are not enough left to fill gaps in the gene sequence, i.e., to join the shorter contigs. The same problem is seen with HMM-GRASPx. Since it utilizes all reads (in protein space) in its simultaneous search and assembly algorithm, short contigs might be caused by different factors in its pipeline such as the re-calibration step where locally extended contigs are merged. Xander, MegaGTA, and MEGAN, on the other hand, are able to assemble longer contigs because they work from all reads in the sample (at the cost of much larger memory usage and CPU time to load all data) and might also have more robust algorithms to maximize contig lengths.

Sensitivity is also of paramount importance. Considering the number of target genes matched with 100% identity, GenSeed-HMM scored highest, matching all target sequences in the synthetic and mock data. SAT-Assembler scored nearly as well, not considering *nifH*. It matched all *nirK* genes in both the synthetic and mock data, all *rplB* genes in the synthetic data, and all but one of the 48 bacterial *rplB* genes in the mock data. HMM-GRASPx did as well for the synthetic data and additionally assembled contigs that matched all *nifH* genes in the synthetic data, which is something SAT-Assembler failed to do. MEGAN did just as well with the synthetic data but matched only 35 *rplB* genes in the mock data and only four of the five *nirK* genes in the mock data. It did the best at matching 16 of the 18 *nifH* genes in the mock data at 100% identity. It is easy to understand MEGAN’s performance at providing 100% matches to the target genes. Because of the way it works, the contigs it produces are essentially genes in NCBI-nr. As long as a gene in NCBI-nr is well represented in the sample, it is what you get back as the contig. This also means that MEGAN is less likely to capture novel gene diversity in environmental samples. Thus, with different datasets, different genes, and identity cutoff, it is difficult to find the tool with highest sensitivity. It is, however, also important to take assembly length into consideration since the sequence length is critical for target gene-based molecular ecology and diversity analyses. After filtering assemblies with length cutoff of 450 bp, Xander and MegaGTA provided the best sensitivity with all three datasets for *rplB* and *nifH*.

Another aspect of assembly quality is the production of non-target sequences, i.e., false positives. All assemblers produced some, but Xander and MegaGTA by far produced the fewest while GenSeed-HMM, MEGAN, and SAT-Assembler produced the most. Some produced by MEGAN were exceedingly long and matched portions of a genome in the synthetic or mock community with 100% identity. MEGAN assembler works by assembling all reads mapped to a GO (in our case) or KEGG category (Huson et al., 2017). We suspect that the production of non-target contigs has to do with how reads are mapped, and possibly with errors in the mapping file that maps NCBI IDs to functional categories in GO.

In most cases, chimeras are to be expected among close relatives from assembly of shotgun data whether gene-targeted or whole genome. MEGAN is the exception here because, as mentioned above, contigs are usually essentially genes or genome segments of what is in NCBI-nr. Our results are therefore somewhat surprising and encouraging. With the exception of SAT-Assembler, nearly all chimeras detected were between the most closely related sequences suggesting accurate taxonomic classification to the genus level.

“How much sequencing do I need” is often the first question asked when designing a metagenomics project. The answer depends on the target species (usually with specific functions) of interest, since it is difficult to estimate the true diversity (Rodriguez and Konstantinidis, 2014; Rodriguez-R et al., 2018) and also costly to sequence deep enough to cover most species in complex environments (Locey and Lennon, 2016). Therefore, sequencing depth estimates based on a target species or function is critical for experiment planning. With our derived model, the relation between the amount of sequencing data and the probability of assembling a contig with at least “M” bp of the target gene with a size of “S” bp from taxa with a relative abundance of “R” can be determined (**Figure 1**). The relative abundance (“R”) can be estimated using common 16s rRNA gene amplicon or qPCR methods. This estimate is a lower bound, since sequencing error, repeats, and micro-heterogeneity among closely related strains could complicate assembly of the target gene.

Because it is difficult to have enough sequencing depth to cover most species in a high diversity sample, follow-up questions are “how many rare members are not assembled” and “how does sequencing depth change the assembled read ratio?” Even though each rare member is only a small percentage of the total community, their sum could be a significant part of the community and thus have a significant role in community function. Missing rare members is an unavoidable problem for all assembly-based methods because there is simply not enough coverage (Guo et al., 2018). There are two cases of rare members: (1) those that are too rare to yield any read coverage and (2) those that have some coverage but not enough to assemble the target gene with minimum length. Here, we focus on the latter. In our soil sample (C1), the number of *rplB* assembled decreased much faster than linear decrease with sequencing depth (**Figure 2**), suggesting that sequencing depth has a strong impact on gene-targeted assembly in diverse communities and thus careful planning on sequencing depth is critical. As an upper bound, the quantity of a targeted gene can be assessed from the number of short reads annotated as the targeted gene without assembly. While this minimizes missing low coverage members, it often includes false positives (low specificity) when there are conserved motifs among protein families. There have been efforts to tackle this problem such as finder function in HMM-GRASPx and ROCKer (Orellana et al., 2017). Also, ROCKer builds gene specific models that set specific sequence similarity score thresholds for different regions of a gene. These kinds of tools can not only improve gene quantification but also could be used as a preprocess step for all above gene-targeted tools, e.g., ROCKer has been shown to improve the accuracy of Xander (Orellana et al., 2017).

All tools reviewed here except MEGAN make use of pHMMs built from reference sequences. The use of pHMMs has clear advantages. It is a faster and more effective way to search gene fragments compared to pairwise alignment as implemented by BLAST or DIAMOND. Additionally, pHMM-based profile search can improve the sensitivity for remotely related protein identification (Eddy, 2009; Zhang et al., 2014; Reyes et al., 2017). The performance of pHMM-based tools, however, is dependent on the quality of the pHMMs used, which in turn is dependent on the appropriateness of the reference sequences used to build them. Ideally, the pHMMs will selectively capture all diversity in the gene family.

The availability of reliable pHMMs may influence the choice of tools used. MEGAN does not require them, and SAT-Assembler is designed to work with pHMMs downloaded from Pfam. Xander (and hence MegaGTA), however, come with a limited set of pHMMs and required reference sequences for finding starting kmers. Instructions are provided for adding capability for additional genes to Xander. The FunGene (Fish et al., 2013) website is provided to help with this task, but knowledge of the gene’s diversity is required. Profile HMMs are built to capture conserved regions (domains) of a gene family, and there is usually enough variation to divide the gene family into sub-groups. If the sequences used to build the pHMM do not include all subgroups of the gene, then not all gene diversity will be captured from metagenomic data. In some cases, as was shown for *nosZ* (Sanford et al., 2012), there is too much diversity to be captured by a single pHMM; hence, multiple models are necessary. Based on our experience, if there is large sequence variation in a gene (<50% identity), then it should be split, and subgroups can be defined based their segregation on a phylogenetic tree. Thus, results are strongly dependent on the care with which the models are built.

Microbial ecologists are interested in comparing microbiomes among environments or treatments with respect to diversity and function. Metagenomic analyses can answer these questions, but the tools used must accurately assemble and quantify target genes in a manner that allows comparisons among samples. Of the tools reviewed here, only Xander and MegaGTA offer this capability directly (**Table S9**). Their search script includes steps for removing chimeras, clustering reads based on a user-defined distance, providing coverage adjusted counts, and taxonomically matching representative sequences to sequences in a database. An additional script is provided to combine this information from multiple samples to create files that may be imported into phyloseq (McMurdie and Holmes, 2013) as a coverage adjusted OTU table, representative sequences, and, with a function in RDPutils (Quensen, 2018), a corresponding taxonomy table. This gives great flexibility for subsequent analyses. MEGAN can also generate OTU tables and ordinate samples based on taxonomy from all reads, but not in a way that the results are based on a particular set of (pathway related) genes. Additionally, the high proportion of false positives we observed with MEGAN makes using its results for comparative analyses of functional genes questionable. Using SAT-Assembler or GenSeed-HMM results to make like comparisons would require writing additional custom scripts. HMM-GRASPx failed to assemble sequences from complex data, and its authors caution that its results are not quantitative. Most tools except Xander and MegaGTA do not have post-assembly diversity analyses across samples, but they can be improved by applying the post-assembly processing method in Xander. Further improvements can be made on Xander and MegaGTA too. Currently, their post-assembly processing method is designed for assembling each sample individually, but not for pooled assembly, which is common practice applied to increase coverage of rare species. Moreover, they do not directly provide a BIOM table that integrates both OTU table and taxonomy information (McDonald et al., 2012) and can be imported into other commonly used microbial diversity analysis tools such as Mothur (Schloss et al., 2009) and QIIME (Caporaso et al., 2010).

We tested the tools under comparable conditions by using default parameters, which by no means are the optimal parameters; especially kmer or overlap size can strongly impact contig length and number and chimera number. We did not try to find the optimal set of parameters for each tool and only adjusted them when a tool performed significantly more poorly than others, i.e., SAT-Assembler produced too many short and chimeric contigs, and we improved its results by increasing the overlap length.

## Summary and Outlook

Gene-targeted assembly offers advantages for metagenome analysis over whole genome assembly and binning because of (1) higher quality assembly (fewer chimera), (2) more extensive recovery of genes of interest (more sensitivity), and (3) faster and less costly analysis of complex communities which also makes these analyses available to a larger set of researchers. It does, however, give up information on gene context and host taxa that come from genome binning. Long-read sequencing, now available but in its infancy, has the potential to make assembly obsolete, but the present high error rates and low capacity make its reliable and routine use some years away. In the meantime, further improvements of gene-targeted tools, some of which are noted above, will help speed the analysis of the now huge metagenomic data in public databases plus the data from even larger sequencing efforts underway.

## Data Availability Statement

Publicly available datasets were analyzed in this study. These data can be found here: https://trace.ncbi.nlm.nih.gov/Traces/sra/?run=SRR606249 and https://trace.ncbi.nlm.nih.gov/Traces/sra/?run=SRR3989263.

## Author Contributions

JG and JQ performed the analyses under the supervision of JT, JC, YS, QW, and CB. All also helped with the analytical approaches and writing of the manuscript.

## Funding

Support for this research was provided by the U.S. Department of Energy, Office of Science, Office of Biological and Environmental Research (Awards DE-FC02-07ER64494 and DE-FG02-99ER62848); National Institute of Environmental Health Science, Award N0172U P42ES004911; and by the National Science Foundation, Awards DBI-1356380, DBI-1759892 and DEB 1637653.

## Conflict of Interest

The authors declare that the research was conducted in the absence of any commercial or financial relationships that could be construed as a potential conflict of interest.

## Abbreviations

pHMM, protein profile hidden Markov model; DBG, *de Bruijn* graph; kmer, subsequence of length k; OTU, operation taxonomic units; Gbp, 1 billion base pairs; GB, 1 billion bytes; *rplB*, the gene encoding 50S ribosomal large subunit L2; *rpsC*, the gene encoding 30S ribosomal small subunit protein S3; *nifH*, the gene encoding nitrogenase reductase; *nirK*, the gene encoding nitrite reductase.
