# Supplementary material for: Review, Evaluation, and Directions for Gene-Targeted Assembly for Ecological Analyses of Metagenomes
Source: Front Genet. 2019 Oct 15;10:957. doi: 10.3389/fgene.2019.00957 (PMC6843070; doi:10.3389/fgene.2019.00957)
Supplement: Supplementary file 1 [file DataSheet_1.pdf]

Supplemental Materials

	Search filter	pHMM	Graph	Post process	Output DNA	Output Protein
Xander	N	Assembly	DBG	Y	Y	Y
MegaGTA	N	Assembly	DBG	Y	Y	Y
SAT	Y	Filter	Overlap	N	Y	N
HMMGRASPx	Y	Filter, assembly	Overlap	N	Y	Y
Genseed-HMM	Y	Filter	NA	N	Y	N
MEGAN	N	NA	Overlap	N	Y	N

Table S1. Overview of gene-targeted tools. Finder: function to filter reads that are fragments of target genes. pHMM: where pHMM is used in algorithms. Post assembly process: steps after assembly to enable diversity analyses and comparison among samples. Y - function is provided. N – function is not provided. Genseed-HMM's assembly graph is "NA" since it depends on the third party tool that was chosen in its pipeline. MEGAN's pHMM is "NA" since it is not pHMM-based tool.

16

ID	Description	Phylum
NZ_CP009257.1	*Acinetobacter baumannii* strain AB030	*Gammaproteobacter*
NZ_CP007793.1	*Azospirillum brasilense* strain Az39	*Alphaproteobacteria*
NZ_CP007794.1	*Azospirillum brasilense* strain Az39 plasmid AbAZ39_p1	*Alphaproteobacteria*
NC_012483.1	*Acidobacterium capsulatum* ATCC 51196	*Acidobacteria*
NZ_CP010415.1	*Azotobacter chroococcum* NCIMB 8003	*Gammaproteobacteria*
NZ_CP010421.1	*Azotobacter chroococcum* NCIMB 8003 plasmid pAcX50f	*Gammaproteobacteria*
NC_012560.1	*Azotobacter vinelandii* DJ	*Gammaproteobacteria*
NC_014638.1	*Bifidobacterium bifidum* PRL2010 chromosome	*Actinobacterium*
NC_004722.1	*Bacillus cereus* ATCC 14579 chromosome	*Firmicutes*
NC_008255.1	*Cytophaga hutchinsonii* ATCC 33406	*Bacteriodetes*
NC_012489.1	*Gemmatimonas aurantiaca* T-27 DNA	*Gemmatimonadetes*
NZ_CP016094.1	*Lacunisphaera limnophila* strain IG16b chromosome	*Verrucomicrobia*
NC_002516.2	*Pseudomonas aeruginosa* PAO1 chromosome	*Gammaproteobacteria*
NZ_CP014784.1	*Pseudomonas alcaligenes* strain NEB 585	*Gammaproteobacteria*

NC_016830.1	*Pseudomonas fluorescens* F113	*Gammaproteobacteria*
NC_009439.1	*Pseudomonas mendocina* ymp	*Gammaproteobacteria*
NC_002947.4	*Pseudomonas putida* KT2440 chromosome	*Gammaproteobacteria*
NZ_CP027543.1	*Pseudomonas stutzeri* strain DW2-1 chromosome	*Gammaproteobacteria*
NC_007005.1	*Pseudomonas syringae pv. syringae* B728a chromosome	*Gammaproteobacteria*

Table S2. Genomes used to create the synthetic data set.

Method	Contigs	Length	Non-target	<97%	97%	98%	99%	100%
Xander	11	585-876	0	3	6/8	5/5	3/3	3/3
MegaGTA	11	585-876	0	3	6/8	5/5	3/3	3/3
HMM-GRASPx[1]	43	66-354	0	14	6/29	6/29	6/27	6/27
HMM-GRASPx[2]	0	>=450	-	-	-	-	-	-
MEGAN[3]	20	204-1,464	11	0	6/9	6/9	6/9	6/7
MEGAN[4]	11	474-1,464	6	0	5/5	5/5	5/5	3/3
SAT-assembler	NF	-	-	-	-	-	-	-
Genseed-HMM[5]	127	33-1360	71	0	6/56	6/56	6/56	6/56
Genseed-HMM[6]	48	453-1360	46	0	2/2	2/2	2/2	2/2

Table S3. BLAST summary for *nifH* contigs assembled from the synthetic data. There were 6 *nifH* sequences present in the synthetic data. HMM-GRASPx[1]- all contigs; HMM-GRASPx[2] – filtered to at least 450 bp in length; MEGAN[3] – all contigs; MEGAN[4] – filtered to at least 450 bp in length. Genseed-HMM[5]: all contigs assembled; Genseed-HMM[6]: contigs were filtered to a minimum length of 450 bp.

Method	Contigs	Length	Non-target	<97%	97%	98%	99%	100%
Xander	2	456-465	0	0	2/2	2/2	2/2	2/2
MegaGTA	2	456-465	0	0	2/2	2/2	2/2	2/2
HMM-GRASPx[1]	21	78-195	0	11	4/10	4/10	4/10	4/10
HMM-GRASPx[2]	0	>=450	-	-	-	-	-	-
MEGAN[3]	8	423-1,497	3	0	4/5	4/5	4/5	4/5
MEGAN[4]	7	567-1497	3	0	4/4	4/4	4/4	4/4
SAT-assembler[5]	53	155-947	46	0	4/7	4/7	4/7	4/7
SAT-assembler[6]	5	460-947	0	0	4/5	4/5	4/5	4/5
Genseed-HMM[7]	29	94-1376	25	0	4/4	4/4	4/4	4/4
Genseed-HMM[8]	18	492-1376	14	0	4/4	4/4	4/4	4/4

Table S4. BLAST summary for *nirK* contigs assembled from the synthetic data. There were 4 *nirK* sequences in the synthetic data. HMM-GRASPx[1]- all contigs; HMM-GRASPx[2] – filtered to at least 450 bp in length; MEGAN[3] – all contigs; MEGAN[4] – filtered to at least 450 bp in length; SAT-Assembler[5] – all contigs; SAT-Assembler[6] – contigs were de-replicated, and filtered to at least 450 bp in length. Genseed-HMM[7]: all contigs assembled; Genseed-HMM[8]: contigs were de-replicated and filtered to a minimum length of 450 bp.

Method	Contigs	Length	Non-target	<97%	97%	98%	99%	100%
Xander	27	537-879	0	5	16/22	16/22	16/22	14/15
MegaGTA	28	537-879	0	5	16/23	16/23	16/23	15/16
MEGAN[1]	68	204-3702	45	1	17/22	17/21	17/21	16/20
MEGAN[2]	39	450-3702	25	1	12/13	12/12	12/12	11/11
SAT-assembler	NF							
Genseed-HMM[3]	439	31-1614	290	6/13	18/136	18/132	18/100	18/100
Genseed-HMM[4]	198	452-1614	184	2/2	11/12	11/12	10/11	10/11

Table S5. BLAST summary for *nifH* contigs assembled from the mock data. MEGAN[1]: all contigs assembled. MEGAN[2]: contigs filtered to a minimum length of 450 bp. NF – not found. Mock data by HMM-GRASPx failed to complete. Genseed-HMM[3]: all contigs assembled; Genseed-HMM[4]: contigs were filtered to a minimum length of 450 bp. There were 18 *nifH* sequences in the strains included in the mock data.

Method	Contigs	Length	Non-target	<97%	97%	98%	99%	100%
Xander	2	621-702	0	0	2/2	2/2	2/2	2/2
MegaGTA	3	507-702	0	0	2/3	2/3	2/3	2/2
MEGAN[1]	30	210-2238	26	0	4/4	4/4	4/4	4/4
MEGAN[2]	13	480-2,238	9	0	4/4	4/4	4/4	4/4
SAT-assemlber[3]	256	60-533	156	0	5/100	5/100	5/98	5/88
SAT-assembler[4]	2	533-533	0	0	1/2	1/2	1/2	0
Genseed-HMM[5]	43	61-1343	35	0	5/8	5/8	5/8	5/8
Genseed-HMM[6]	27	509-1343	21	0	5/6	5/6	5/6	4/5

Table S6. BLAST summary for *nirK* contigs assembled from the mock data. MEGAN[1]: all contigs assembled. MEGAN[2]: contigs filtered to a minimum length of 450 bp. SAT-Assembler[3]: all contigs assembled. SAT-Assembler[4]: contigs were de-replicated, duplicates removed, and filtered to a minimum length of 450 bp. There were 5 *nirK* sequences in the strains included in the mock data.

Assembler	Contigs	Chimera	% Chimera	Same Genus
Xander	28	12	42.9	11
MegaGTA	28	12	42.9	11
SAT-assembler	176	79	44.9	57
SAT-assembler-450	106	74	69.8	54
MEGAN	55	0	0.0	NA
MEGAN-450	20	0	0.0	NA
Genseed-HMM	97	0	0.0	NA
Genseed-HMM-450	9	0	0.0	NA

Table S7. UCHIME summary for *rplB* contigs assembled from the synthetic data.

Assembler	Contigs	Chimera	% Chimera	Same Genus
Xander	95	5	5.3	5
MegaGTA	94	5	5.3	5
SAT-assembler	2,765	104	3.8	60
SAT-450	61	18	29.5	13
MEGAN	93	0	0	NA
MEGAN-450	50	0	0	NA
Genseed-HMM	408	1	0.2	1
Genseed-HMM-450	44	0	0	NA

Table S8. UCHIME summary for *rplB* contigs assembled from the mock data.

Assembler	Contigs	Chimera	% Chimera	Same Genus
Xander	11	5	45.5	5
MegaGTA	11	5	45.5	5
MEGAN	20	2	10.0	2
MEGAN-450	11	2	18.2	2
Genseed-HMM	127	0	0	NA
Genseed-HMM -450	48	0	0	NA

Table S9. UCHIME summary for *nifH* contigs assembled from the synthetic data.
